# Shaping mindful citizens: Practitioners’ motivations and aspirations for mindfulness in education

**DOI:** 10.1177/13634593231179024

**Published:** 2023-09-19

**Authors:** Peter J Hemming

**Affiliations:** University of Surrey, UK

**Keywords:** ageing and life course, complementary and alternative medicine, ethnography, health, mental health, quality of life

## Abstract

Mindfulness meditation has enjoyed growing popularity in the UK over the last few decades and is increasingly found in many educational settings. To date, existing empirical research on mindfulness in education has focused primarily on its efficacy, rather than more sociological concerns. This article draws on qualitative data from a major research study entitled ‘Mapping Mindfulness in the UK’ to investigate the motivations and aspirations of mindfulness practitioners for promoting and delivering mindfulness in educational contexts. The analysis argues that some of the existing theoretical critiques of mindfulness as a neo-liberalising self-technology are too reductive and do not take adequate account of the views and experiences of practitioners. For participants in this study, mindfulness in education was more than an individualised self-help therapeutic tool, but was instead a uniquely versatile practice, representing multiple possibilities for individuals and society. The research makes significant contributions to several fields of sociological inquiry, including on mindfulness, mental health and wellbeing, and education and citizenship.

## Introduction

Mindfulness has enjoyed growing popularity in the UK over the last few decades. According to a 2018 survey, 15% of adults in Britain (almost 8 million) have learnt how to practice mindfulness meditation (Simonsson et al., 2021). Once confined to mainly Buddhist or clinical settings, mindfulness has moved into the public sphere, and can be found in a range of contexts, from parliaments to prisons, and workplaces to self-help apps. Education is no exception, with mindfulness playing an increasingly prominent role in the everyday life of many schools, colleges and universities (e.g. see Swain, 2016). Although not a compulsory part of any state-directed curriculum in the UK, the practice often features in education through wellbeing initiatives, situated alongside, or embedded within, formal curricula, and delivered by existing staff or visiting practitioners. This process has been supported by several policy developments, such as the publication of the ‘Mindful Nation UK’ report by the Mindfulness All-Party Parliamentary Group (2015), which recommended a greater role for mindfulness in education.

To date, existing empirical studies on mindfulness in education have primarily focused on its efficacy, often demonstrating moderate benefits for student mental health and wellbeing (e.g. Carsley et al., 2018; Weare, 2013), but sometimes reporting less positive results (e.g. Montero-Marin et al., 2022). However, there is less research that addresses sociological questions such as the scale and nature of its presence, the motivations and aspirations of its proponents, and the views and experiences of those who encounter it. Previous empirical studies of this kind are relatively limited in number and have mainly been concerned with qualitative evaluation of specific mindfulness initiatives, implementation case studies, or the experiences of teachers and students from a health and wellbeing perspective (e.g. Hutchinson et al., 2018; Norton and Griffith, 2020; Wilde et al., 2019). There is thus a need for larger-scale research that takes a critical, sociological approach to these issues.

This article draws on qualitative data from a major research study, entitled ‘Mapping Mindfulness in the UK’, to investigate the views and experiences of mindfulness practitioners, trainers and advocates regarding the role of mindfulness in education (broadly defined). It explores their stated motivations for promoting and delivering mindfulness in educational settings and examines their aspirations for students who receive the training. The findings are discussed in the context of wider socio-cultural debates about mindfulness, including its association with therapeutic and character education, and its relationship with capitalism and neo-liberalism. The research makes significant contributions to several fields of sociological inquiry, by advancing new perspectives on mindfulness and education, mental health and wellbeing, and citizenship and governance.

## Mindfulness, education and citizenship

Mindfulness is a word with many meanings, and even Buddhist forms of mindfulness meditation are generally recognised as a product of cross-cultural exchange and transformation, shaped through particular social and historical contexts, and comprising a variety of definitions across different traditions (Wilson, 2014). Whilst its Buddhist roots are not explicitly denied, modern mindfulness is typically framed as a secular practice, accessible to a range of participants, whatever their religious or non-religious background (Sun, 2014). Kabat-Zinn (2005), often considered the founder of modern mindfulness for his development of Mindfulness-Based Stress Reduction (MBSR) within clinical settings in 1980s Massachusetts, defines mindfulness as ‘moment-to-moment, non-judgemental awareness’ (p. 58), and as an innate, universal capacity that all humans possess. Mindfulness is touted as a versatile self-help technique that can support individuals to achieve a variety of goals, including stress relief, pain reduction, treatment of anxiety and depression and enhanced concentration (e.g. Cho, 2016). In an educational context, students are most likely to encounter mindfulness through directed activities that introduce them to certain meditation techniques. These can take a variety of forms, from a few minutes of focused breathing at the start of a learning session, to a more substantial programme of training in contemplative practices such as body and sense awareness, walking meditation and mindful yoga.

The main impetus for mindfulness in education centres primarily on concerns about mental and emotional ill-health amongst the young. Drawing on the idea that stages of the life-course can be understood as socially constructed rather than biologically given, sociologists have long documented the persistent notion that childhood and youth are somehow in crisis (Kehily, 2010). The most recent iterations of this trend have claimed that children and young people are experiencing increasing levels of unhappiness, stress and anxiety, due to a mixture of excess consumerism, technology use, academic testing, and unhealthy diets, alongside a lack of opportunities for exercise, outdoor play, restful sleep, and meaningful social interaction (Palmer, 2015). Whilst recent figures do show increases in the number of children and young people being diagnosed with mental health conditions (e.g. Peytrignet et al., 2022), it is difficult to establish with any certainty that wellbeing amongst the young is indeed declining, as such figures could instead indicate growing levels of awareness and diagnoses, rather than substantive changes.

The above concerns have fuelled the growth of various interventions in educational settings, aimed at promoting the emotional wellbeing and emotional literacy of children and young people, including circle time, buddy schemes and nurture groups, with mindfulness often cited as another example (Ecclestone and Hayes, 2009). Such developments can be viewed through the lens of character education, which has long been an important feature of education in the UK, from the establishment of mass schooling in the late 19th Century, intended to ‘civilise’ the ‘unruly’ working classes, to the post-war emphasis on moral character, as a response to the atrocities of World War II (Arthur, 2010). However, since the turn of the 21st Century, there has been a renewed focus on character and citizenship education, with increasing attention directed towards emotional aspects of students’ development (Walker et al., 2015). According to Wright (2014), this growth in ‘therapeutic education’ can only be fully understood when situated within wider streams of scholarship that highlight the increasing role of psychology and therapeutic cultures for citizens’ everyday lives, identities and sense of self.

Placed within this broader frame of therapeutic and character education, mindfulness can be understood as a type of education for citizenship, with the potential to contribute to various educational goals and agendas, including those associated with neo-liberalism. Ecclestone and Hayes (2009) have argued that therapeutic education constructs the human subject as fragile and vulnerable, positioning students as in constant need of affective interventions and privileging the development of ‘soft skills’ over traditional knowledge-based curricula. Brunila (2012) contends that it encourages an excessive focus on the self, encouraging students to view societal issues, such as unemployment or educational failure as personal problems, for which they are individually responsible and to which they are expected to show appropriate levels of emotional resilience. Gagen (2015) suggests that social and emotional learning programmes reframe education for citizenship to encompass the cultivation of emotional competence alongside political literacy, such as the development of self-awareness, anger management and impulse control, to encourage self-restraint and maximise productivity. Such critiques draw implicitly on Foucault’s (1982, 2008) concepts of ‘pastoral power’, which refers to the modern evolution of sovereign and disciplinary power into a form that positions individuals as responsible for the shaping of their own selves, and ‘governmentality’, which signifies the governing strategies and procedures that seek to guide citizens’ conduct, through the creation of social and moral environments that encourage individuals to behave in certain ways, but of their own volition.

Foucauldian influences also feature very prominently in socio-cultural critiques that focus more specifically on mindfulness and its role in wider society, many of which have also been applied to education (Primdahl, 2022). On the relationship between mindfulness and neo-liberalism, proponents point to the benefits that the practice can afford for individual wellbeing, and its accessible and universal nature as a secular therapeutic technique for combatting the stresses of life in modern societies (e.g. Gelles, 2015). In the context of education, Hyland (2011) argues that mindfulness can enrich learning and act as an antidote to the neo-liberal obsession with standards, targets and testing. However, scholars such as Purser (2019) have employed the phrase ‘McMindfulness’ to describe the ways that mindfulness may be used to individualise the challenges associated with modern life. Rather than the authorities working to address the toxic work practices associated with the contemporary workplace, or the precarious employment conditions of the neo-liberal labour market, it is argued that individuals are instead encouraged to take responsibility for managing their own wellbeing. Mindfulness can thus be understood as a mechanism of neo-liberal governance that recruits the individual as a willing participant in their own regulation, and hence supports and enables the continuation of existing economic and political structures.^1^ Reveley (2016) appraises mindfulness provision in schools through this same lens, viewing it as a neo-liberalising self-technology that effectively medicalises education by widening the definition of illness to include everyday emotional impulses, which students are taught to manage as developing entrepreneurial citizens.

A closely related critique pertains to the relationship between mindfulness, capitalism and consumerism, and the extent to which mindfulness may be subject to appropriation by certain ideological agendas (also sometimes referred to as ‘McMindfulness’). Carrette and King (2004) have previously discussed the ways in which spiritual practices can become hijacked by corporate interests for the maximisation of profit and the furthering of capitalist imperatives. The construction of mindfulness as a universal, self-help technique, creates the potential for it to be marketed as a commodity across a wide range of contexts, from mindful colouring to parenting, and mindful leadership to cookery (Walsh, 2016). Whilst proponents of such products and services might argue they are beneficial, some of these more instrumental applications are quite far removed from the wellbeing imperatives discussed previously, and certainly from associated Buddhist philosophies. For example, mindfulness has been used to support ideologies of warfare, with the practice delivered in military settings to help service personnel focus and concentrate during combat (Stone, 2014).^2^ There are also concerns that mindfulness risks the furthering of capitalist educational objectives, such as compliant behaviour, attention resilience, and optimal performance amongst students (Forbes, 2019).

According to some observers, the precise relationship between mindfulness, neo-liberalism and capitalism can depend on the type of mindfulness under investigation. McCaw (2020) makes a distinction between ‘thin’ mindfulness (an ethically neutral, psychological technique for self-improvement) and ‘thick’ mindfulness (an ethically grounded practice for transformation, rooted in Buddhist or other radical ontologies). He argues that mindfulness initiatives in education typically draw on the former, but that the latter is required for meaningful organisational or societal change, driven through the cumulative behaviours of individuals, Similarly, Ergas (2019) draws the distinction between mindfulness *in* education and mindfulness *as* education, where the former is aligned with *socialisation* of children and young people into the existing social order, whilst the latter aims for *acculturation* of students and the reformation of society through wisdom, knowledge and virtue. Such analyses further highlight the potential and variable influences that mindful practices might have on the shaping of students as future citizens within educational contexts - a central concern of the present study.

## Methodology

The data in this article forms part of a larger study, entitled ‘Mapping Mindfulness in the UK’, which aimed to provide a detailed account of the ‘who’, ‘what’, ‘where’, ‘why’ and ‘how’ of UK mindfulness provision, across a variety of sectors, and evaluate this in relation to wider socio-cultural trends. The project included a nationwide online survey of 768 self-defined, trained teachers of mindfulness, distributed through practitioner networks, along with 82 in-depth online interviews (20–75 minutes duration, averaging 45 minutes) and 4 online discussion groups (comprising 4–7 members and lasting 110–115 minutes) with selected mindfulness practitioners, trainers and advocates. Ethnographic fieldwork also took place in 28 settings across the UK, including larger-scale public conferences and networking events and smaller-scale private classes and training courses, representing a range of sectors. Fieldwork lasted anywhere between 1.5 hours and 8 days at each setting, and observations focused primarily on the actions of practitioners rather than their client groups. Data was generated between 2017 and 2021.

Informed consent was sought from all survey respondents, interviewees, discussion group members, event organisers in larger-scale public settings, and mindfulness teachers in smaller-scale private settings (where researcher presence was also publicised to attendees/students, as well as parents/carers in the case of school-based fieldwork). All participants have been attributed pseudonyms to maintain anonymity. The study was granted approval from the ethics committee at Cardiff University’s School of Social Sciences.

The present article draws on a subsection of the larger dataset, focusing on qualitative material holding relevance for the education sector, to investigate the motivations and aspirations of mindfulness practitioners, trainers and advocates for promoting and delivering mindfulness in educational settings. This includes data from the interviews, open-ended survey questions, and one of the discussion groups that focused specifically on education. The above data is supported with brief reference to 8 of the ethnographic case-studies that were most relevant to education, as detailed in Table 1. All qualitative data pertaining to education was analysed thematically, through an interpretivist framework.

**Table 1.** Ethnographic case-studies.

	Ethnographic field-site	Duration	Year	Approx. no. of attendees
Site A	Conference for education professionals interested in mindfulness in school contexts	1 day	2018	800
Site B	Teacher training course for a popular mindfulness programme in primary schools	3 days	2019	25
Site C	Policy seminar and networking event on promoting mindfulness in UK education	1.5 hours	2018	150
Site D	School-based observations of a popular mindfulness programme delivered to children aged 8–11	4 × 1 hour	2019	20–30 × 4 classes
Site E	Conference of an organisation providing mindfulness course and retreats	4 days	2019	150
Site F	Exhibition and trade show attended by mindfulness providers and members of the public	2 days	2018	2000
Site G/H	Policy and networking conferences on promoting mindfulness in Wales	2 × 1 day	2017 2019	30 100

The interview sample of 82 mindfulness practitioners, trainers and advocates comprised 44 women and 38 men, working in a variety of fields, including health, psychology/therapy, coaching, education, workplace training, religious/spiritual settings, politics/policy, technology, social work/addiction, criminal justice, mindful products, social change initiatives, and the general public. The discussion group comprised 6 participants, including 4 women and 2 men, all of whom were leading figures in the mindfulness and education sector, working with schools, families, higher education and youth work. As such, participants referred to a range of different forms of mindfulness teaching and types of educational setting and had various levels of personal involvement with mindfulness in education.

The survey sample included representation from across the UK, encompassing those who had taught mindfulness in England (69%), Scotland (12%), Wales (4%), Northern Ireland (<1%) and more than one UK nation (15%). The demographics of the sample were predominantly female (70%), white (89%), heterosexual (82%), non-disabled (87%), middle-aged at 40–64 years (80%), and highly educated to postgraduate level (67%). 42% of respondents reported they had taught mindfulness in the education sector (alongside other sectors in many instances) and 21% had trained in programmes that were education related. These included courses run by the Mindfulness in Schools Project,^3^ such as *dot B, Paws B* and *dot B Foundations*, as well as Youth Mindfulness^4^ (including *Youth Mindfulness Kids*). Other education related courses included *The Present*,^5^ *Myriad Teachers’ Programme*,^6^ *Mindful Schools*,^7^ *Mind Up*^8^ and unspecified mindful parenting courses.

## Mindful motivations

At the most basic level, many participants were motivated by a desire to help children and young people and to make a difference to their lives, through teaching mindfulness in educational contexts. Some had experienced personal benefits from their own mindfulness practice, usually in terms of improved mental health and wellbeing, and wanted to share these same benefits with others. The theme of ‘making a difference’ was also prominent at conference events including Site A and H.

*Survey Respondent 29*:

I want to share the practice so that others may also experience its benefits. I hope that by teaching the children at school some of them may find it helpful and want to continue to develop their own practice later in their life.

Beyond this, mindfulness was viewed as a vehicle for addressing what was perceived to be a widespread and growing problem. Many of the interview participants, as well as speakers at the conference and training events (Sites A, B, C, E and F), floated the idea that the UK was currently facing a crisis of mental ill health. They discussed how contemporary life was becoming so busy and stressed that unhealthy ways of living had increasingly become the norm. There was a sense that the young were particularly impacted by this trend, with children and young people frequently constructed as being under too much pressure.

*Interview Participant 37*:

If you look at Child and Adolescent Mental Health Services in the NHS [National Health Service], they’re stressed to bursting and the waiting lists are fantastically long and the distress that young people are experiencing is very, very widespread.

For some participants, specific concerns were raised about the negative effects that new technologies and social media might have on the mental health of children and young people, such as overstimulation (see Palmer, 2015). For others, a lack of wellbeing went far beyond these concerns and stemmed from more fundamental issues associated with life in ‘toxic’ modern societies, including consumerism, inequality, ethical deficiencies, and the environmental crisis. Those participants who were interviewed during the COVID-19 pandemic, felt the experiences of lockdown could only further exacerbate these pressures.

*Interview Participant 59*:

We’re creating a soulless world [. . .] We’re very effectively killing it by society and technology, greed, you know, rampant overstimulation of our nervous system, every war that’s going on [. . .]. We’ve all started to realise what the impact of the internet, social media, family breakdown, loss of community, loss of a sense of belonging. And mindfulness fills that hole very effectively.

Many participants expressed the view that current UK education systems were part of the problem, citing concerns with neo-liberal policies, such as the over-emphasis on standards, targets and testing (see Hyland, 2011). This was a message that was also prevalent from speakers at conference events such as Site A and E, who talked about the need for an approach to education that focused more on wellbeing. Some participants cited a growing recognition that change was required to promote better mental health for children and young people but also improve the wellbeing of teachers and parents.

*Survey Respondent 96*:

More people are so stressed that they need a new way forwards. Schools are ridiculous in their computer-driven expectations of staff and students - it’s becoming a toxic environment.

Although there was widespread agreement that the perceived decline in the mental health and wellbeing of children and young people was rooted in the problematic nature of modern society and education systems, there was some divergence in participants’ views on how mindfulness could help address this. Some emphasised helping individual students within the status quo, whereas others saw mindfulness as a vehicle for wider collective change (see Ergas, 2019), as explored in the following sections.

## Mindful aspirations: Individual

In the context of the above concerns, mindfulness was often purported as a useful approach for promoting the wellbeing of children and young people, in some cases, as part of a wider suite of initiatives (see Ecclestone and Hayes, 2009). Many participants felt that mindfulness could give children and young people the individual skills and self-help tools to cope with problems and challenges they might encounter in life, thus helping to improve their overall wellbeing. Some also claimed it was an effective way to reduce mental health suffering.

*Survey Respondent 290*:

I think if you can be taught this skill at a young age, it may help children manage difficult periods of their lives as well as appreciate the normal everyday aspects too.

Many participants stressed the potential of mindfulness to offer a preventative approach to wellbeing in younger age rather than trying to solve mental health problems after they had already developed. Others identified specific mental health conditions amongst children and young people they felt mindfulness could alleviate. At the conference for education professionals (Site A), there were many testaments from children, young people, parents, and teachers who said that mindfulness had changed their life by helping them to overcome experiences of stress, anxiety, depression, fear or trauma. The teacher at the school-based observations (Site D) reported that mindfulness had made a real difference to students who had experienced mental health challenges.

*Survey Respondent 83*:

Mindfulness offers the potential for a preventative-based model focused on improving well-being rather [than] treating difficulties per se. Which is why I am pleased about the development of [popular mindfulness programme for schools]. The younger people start to look after their emotional well-being, the better.

Despite a major focus on the wellbeing benefits that mindfulness purportedly offered, another theme was its educational benefits. These goals tended to be more instrumental, given they also aligned with the priorities of educational institutions. Mindfulness was reported to help with learning and concentration, develop creativity and improve performance. This idea was promoted by one of the speakers at the conference for education professionals (Site A), who talked about nurturing skills for a future world. These ‘instrumental’ benefits were viewed by a few of the participants, either positively or negatively, as part of a wider cultural trend of self-improvement (see Wright, 2014).

*Survey Respondent 618*:

Students and staff often come to mindfulness to help with concentration and attention as well. Students working in health-related areas also find that they want to be more present in their work, particularly with their patients or clients.

The power of mindfulness to encourage calmness in students and hence facilitate learning was also mentioned quite frequently. These benefits were often emphasised by teacher and student speakers at events where mindfulness in education was promoted, including Sites A, B, C, F and H. Some participants were explicit about how mindfulness could help improve student behaviour, through its reported ability to develop emotional literacy and help children and young people to manage their emotions, in line with the goals of therapeutic education (Gagen, 2015). Mindfulness was often cited as something that was especially useful for certain groups of students, such as those with special education needs, or those experiencing problems. These students were the focus of some discussion at the teacher training course (Site B) and during the school-based observations (Site D), where mindfulness was reported to have helped children with social and emotional issues.

*Education Discussion Group Participant B*:

A lot of it is emotional regulation and things like that. [. . .] They’re saying, well, you got a problem with this, can mindfulness help, you know, we want to help students behave better. [. . .] So, the young people themselves, managing themselves within the school context, rather than it being, you know, staff telling them what to do and sort of external discipline structure.

Some participants went beyond wellbeing and instrumental concerns, describing mindfulness as something that could lead to individual human flourishing. In this sense, it was closely aligned with current dominant discourses in character education, which emphasise flourishing and positive psychology (Walker et al., 2015). This was reflected at the teacher training course (Site B), where the promotion of human flourishing was mentioned as one of the aims of mindfulness in education. A few participants also described how mindfulness could tap into fundamental aspects of what it means to be human.

*Interview Participant 26*

I think it’s really important in some shape or form that [mindfulness] is embedded in our schools. Schools can’t be neutral places. You know, we have to engage with our children about what, about what it is to [be] human. And we’ve got to find ways of doing that.

Practitioners thus cited a range of aspirations for students and what they might individually gain from learning and practicing mindfulness, some of which could be considered consistent with critiques of mindfulness and therapeutic education and their relationship to neo-liberal forms of citizenship (e.g. Ecclestone and Hayes, 2009; Forbes, 2019; Reveley, 2016). However, the competing constructions of children and young people as fragile, vulnerable and in need of support to cope with modern life, or as independently productive, focused and self-regulating within a capitalist economy, are somewhat contradictory, and highlight tensions in the original critiques. Moreover, some of the participants’ aspirations concerning human flourishing draw more on liberal humanist models of citizenship than neo-liberal ones and begin to point to alternative possibilities for understanding mindfulness through a social and educational lens.

## Mindful aspirations: Collective

Despite the focus on individual benefits to children and young people, there were some participants who were more sceptical about this individualised approach. There was a feeling that education providers, in the current policy climate, were fundamentally unsuited to developing mindfulness and contemplative practices because of their focus on instrumental outcomes. Participants felt that using mindfulness to try and ‘solve’ particular problems in educational settings or support specific groups of ‘difficult’ students could run the risk of undermining its wider potential. Interviewees talked about the dangers of using mindfulness as a ‘sticking plaster’, rather than developing a more holistic approach that could influence fundamental changes to the cultures of educational institutions (see McCaw, 2020).

*Interview Participant 64*:

Most of the schools that, well, particularly the state schools, they bring you in to solve a problem, rather than embrace it as part of their culture. [. . .] Yeah, they’re bringing you in because they’ve heard it’s a good thing that they don’t really know much about it or understand it. Or they think it might be good for a particular group of students who they have difficulty with and they want to support them.

Others voiced similar concerns but directed at a larger scale – that mindfulness was being used to ‘paper over’ wider systemic problems in education or in society more generally, which could be dangerous if it meant those broader problems were not being addressed. These views somewhat aligned with the McMindfulness critique (Purser, 2019) and the fear that students were being asked to shoulder the consequences of wider structural issues. Implicit in these accounts was an acknowledgement of persistent social inequalities and the potential role of mindfulness in supporting their perpetuation if used uncritically.

*Interview Participant 37*:

There’s a danger that [mindfulness] could actually facilitate a kind of dissociation, you know, so that [. . .] they can actually use the capacity for directed attention to ignore certain suffering in their own experience or certain suffering in the world. [. . .] And so, what I see is mindfulness in education, not in primary schools so much but more in high schools, is being used as a band aid to patch up a system that I think is woefully mismatched to the needs of our world.

Such critiques formed the basis of alternative aspirations that went beyond the individual and focused on groups, communities and wider society, challenging the notion of mindfulness as an individualised practice (e.g. Reveley, 2016). The first of these was the idea that mindfulness could facilitate students connecting with others around them, particularly within educational settings. The practice was reported to be helpful in strengthening relationships between adults and children and young people, whether personal or professional in nature. This purportedly offered the potential to provide a safe space and develop a strong sense of community, including in educational contexts where young people may have previously struggled to express themselves.

*Interview Participant 37*:

I think we need the spaces for young people to come together in groups that are small enough where they can be safe. [. . .] So, with the kids’ program, what’s wonderful about the kids’ program is when that’s taught by a classroom teacher it provides a basis to grow a community of mindfulness. To engage, to have ongoing practice actually change the dynamic of relationships.

A related theme was the potential of mindfulness to cultivate kindness, compassion, and empathy amongst students. Some participants discussed involvement in mindfulness and character education programmes that were overtly aiming to foster such qualities, aligned with wider trends that emphasise virtue-based citizenship and moral education (Walker et al., 2015). A few participants emphasised the potential for individual kindness, compassion and empathy shaped through mindfulness to cumulatively influence institutions and wider society.

*Education Discussion Group Participant B*:

I really feel that mindfulness could perhaps, open up a whole raft of possibilities for young people once they’ve left education. So, when they go off into the business world and into politics, that they might be better informed, possibly more compassionate, more aware, with a sense of greater empathy.

A further discourse emphasised the potential for positive social change and the shaping of a better world, enacted through widespread individual changes in behaviour. Participants felt that expanding the reach of mindfulness in education could help with this mission, through the promise of future ‘mindful’ generations. Such ambitious aims were espoused at some of the conference and exhibition events, including Site A and F, where contributors discussed cultivating positive values and eliminating violence in society. A few participants also went into more detail about the kinds of progressive social change they felt mindfulness could promote, including peace, equality, social justice and environmental sustainability. Such aspirations closely align with the goals of the Buddhist-inspired ‘Contemplative Elite’, as described by Kucinskas (2019) in her study on the rise of mindfulness in the US.

*Survey Respondent 603*:

I hope that mindfulness helps us achieve a solid ethical base based on true human needs, not commercially driven interests, that it starts to help challenge the dominant ethic of individual self-interest, ‘growth’, living for tomorrow, consumption, materialism and waste – and help give rise to a more compassionate and caring society that might actually keep our planet alive by encouraging us to get a hold of our greed and impatience, and slow down and cultivate what actually matters.

Some participants therefore perceived mindfulness to offer much more than a way to enhance wellbeing through individual self-help techniques and to promote instrumental educational aims. Instead, it promised new ways of community living and even large-scale social change. It offered the potential to shape more compassionate, connected and socially aware citizens through a type of wide-reaching character education, constructing students as empowered, rather than vulnerable or compliant, and envisaging more collectivist forms of citizenship. Such aspirations are quite far removed from critiques of mindfulness in education, which tend to focus on how it may work to further, rather than combat, the interests of capitalism and neo-liberalism (e.g. Forbes, 2019).

## Multiple possibilities

The findings above reveal so many different motivations and aspirations for mindfulness in education that the field appeared to be seriously lacking in coherence. This was evident in discussions about how to market mindfulness to education providers, where the main approach seemed to involve highlighting as many potential benefits as possible, as well as the contradictory ways that mindfulness was discussed at events such as the conference for education professionals (Site A). Key distinctions often surfaced through differences in the primary motivations and aspirations of teaching practitioners and those of the educational institutions where they taught, which tended to be more instrumental in nature.

*Interview Participant 10*:

We have a society which is absolutely addicted to the capacity to measure things. So, if you’re going to spend money developing resources to put mindfulness in schools, you want a measurable outcome. In terms of results, behaviour, attendance. That’s right. And that in itself makes it extraordinarily self-limiting, and I know this is frustrating for people.

Most participants did not, however, view this lack of coherence as a fundamental problem. Instead, mindfulness was constructed as something unique in its ability to shapeshift and find a role within different and potentially competing educational agendas (see also Stanley, 2019). When asked about these diverse aims, one of the discussion group participants described a spiral that promoted the same essential mindful qualities at a shallower or deeper level as part of an ongoing learning process. At one of the policy and networking conferences in Wales (Site H), the potential for mindfulness to help students at different levels was expressed through the words ‘cope’, ‘connect’, ‘flourish’ and ‘empower change’.

*Education Discussion Group Participant C*:

Mindfulness is almost, it can almost shapeshift if you like, it’s this almost formless, it can become so many different things, or it can give rise to so many different things. [. . .] Perhaps other wellbeing practices or other spiritual practices may have a limited scope, whereas mindfulness seems to lend itself to this ongoing unfolding into new possibility.

Diverse and sometimes competing goals for the promotion and delivery of mindfulness in education therefore appeared to find a way to co-exist but also created a space for possible tension and contradiction. Sauerborn et al. (2022) paint a similar picture in their analysis of adult mindfulness courses in Germany, highlighting the potentially paradoxical nature of the multiple promises associated with the practice. These findings point to important implications for the kinds of ‘mindful citizens’ that may be shaped within educational contexts through processes of socialisation or acculturation (Ergas, 2019), as discussed further in the final section.

## Discussion and conclusion

This article has drawn on a wide range of qualitative data from the ‘Mapping Mindfulness in the UK’ study to investigate the views and experiences of mindfulness practitioners, trainers and advocates, regarding their motivations and aspirations for promoting and delivering mindfulness in educational settings. In doing so, it has contributed some much-needed empirical research to sociological debates surrounding mindfulness and education, mental health and wellbeing, and citizenship and governance. The study found that most participants were motivated by a desire to help children and young people and to make a difference to their lives, and many framed this as a response to a perceived crisis in mental health and wellbeing. Whilst some practitioners felt these issues could be addressed by helping individuals to use mindfulness to cope and succeed within the existing social order, others felt mindfulness could have a role in changing it, through the development of connection and compassion in students, and cumulative influences on institutions, communities and society (see Table 2 for a summary). However, these two sets of aspirations were not mutually exclusive, with some practitioners viewing them as complementary rather than contradictory. This group viewed mindfulness as a uniquely versatile practice that could be accessed at several different levels as part of an ongoing process, and thus achieve a variety of diverse goals.

**Table 2.** Summary of participant aspirations.

Individual	Collective
Self-help tool for wellbeing – *preventative approach, specific mental health challenges*	Facilitating connection – *child/youth-adult relationships, sense of community*
Educational benefits – *learning, concentration, creativity, performance*	Cultivating kindness, compassion, empathy – *character education, cumulative effects*
Improved behaviour – *calmness, emotional regulation, specific student groups*	Potential for social change – *positive values, peace, equality, environmental sustainability*
Human flourishing – *holistic, experiential, spiritual*	

For the participants in this study, mindfulness in education was about more than just self-help therapeutics. There was clear support for an ethically informed ‘thick’ version of mindfulness that could contribute to education for enculturation (rather than merely socialisation) and offer the potential for both individual and collective transformation (Ergas, 2019; McCaw, 2020). For some practitioners, action was required to help individuals respond to the fast-paced, technologically obsessed, and stressful nature of modern life, but for others, their ambitions reached further, reflecting a desire to combat the excesses of capitalist societies, and tackle inequality, conflict and the environmental crisis. Many participants were fully aware of critiques such as the ‘McMindfulness’ thesis and were keen to stress the limitations of current education systems and the need for reform, rather than using mindfulness as a ‘sticking plaster’ to try and ‘paper over’ certain problems. However, there was also little appetite to abandon or ignore the more instrumental goals of mindfulness, particularly if these could offer benefits to students and/or facilitate access to educational establishments, offering a potential gateway to something more substantial.

Despite their wide array of motivations and aspirations, practitioners appeared united in their wish for change and transformation, whether individual or collective, in education or in wider society. However, the specifics of the desired changes or details on how they might be achieved through mindfulness were usually expressed in quite vague terms. Mindfulness seemed to function as a carrier for diverse hopes and dreams for the future, and as a focal point for alternative and competing visions of education, citizenship and society. It acted as a kind of glue that bound together individuals and groups with disparate aims and ambitions, united in the view that children and young people represented the future, and that early mindful interventions could create positive change. Following Sauerborn et al. (2022), it could be argued that this is one of the reasons for the popularity of mindfulness in education, as well as more broadly. In an era of polarisation, where education has become highly politicised and embroiled within ongoing culture wars, perhaps mindfulness offers an opportunity for consensus within an otherwise fragmented educational landscape. Given everyone can agree on the fact they want to make things better for children and young people, a lack of specificity about exactly *how* things might be improved through mindfulness may act as a useful mechanism for ensuring continuing and widespread support for the practice.

An alternative interpretation understands participants as proactively entrusting the positive futures they imagined and hoped for to the students themselves through their teaching of mindfulness, thus foregrounding their emerging position as social actors. Whilst it is true that many participants appeared to take as their starting point an understanding of children and young people as fragile, vulnerable and in need of mental health and wellbeing support, students were also variously imagined as independent, productive and self-regulating, and/or as connected, compassionate and empowered. Whether competent in managing their own emotional wellbeing, demonstrating proficiency in learning, fostering relationships and community, exploring their own humanity, or furthering wider social change, children and young people were understood as agentic beings, either in the here and now, or as future adult citizens. These constructions offer a more positive and optimistic view of the potential of the young than typically reflected in the critiques of therapeutic education (e.g. Ecclestone and Hayes, 2009), and, whilst not rejecting or minimising its key concerns, move beyond the notion of childhood and youth as in perpetual crisis (see Kehily, 2010).

It would be unfair to claim that ‘McMindfulness’ and related critiques of therapeutic education are completely deterministic. However, they nevertheless portray individuals, to a certain degree, as cultural dupes, relying on therapeutic self-help techniques to willingly participate in the maintenance of the social and political structures that are the source of their original difficulties. Whilst these theories are generally underpinned by Foucauldian concepts of ‘governmentality’ and ‘pastoral power’, Foucault (1998: 95) also insisted that ‘where there is power, there is resistance, and yet, or rather consequently, this resistance is never in a position of exteriority in relation to power’. Some of the practitioners in this present study, although partly enmeshed within the structures of education and its relationship to political authority, were nevertheless using their positions to renegotiate and move beyond the more instrumental versions of mindfulness that were of most interest to educational establishments, and instead pursue diverse agendas that departed from neo-liberal and capitalist imperatives. In turn, they believed they were empowering their students to draw on their own agency to contribute to the shaping of alternative social and moral environments through their individual mindful behaviours and practices – a process that could lead to a myriad of possible futures.

The question remains, however, as to the extent to which practitioners were successful in achieving their diverse aspirations. Just because many of the participants were not aiming for neo-liberal effects does not necessarily mean they did not still occur in practice. Multiple aims are likely to lead to tensions, contradictions and uneven effects and if the purpose of mindfulness in education is unclear, this could result in the conveying of mixed messages to students through inconsistent approaches to teaching the practice. A lack of focus and transparency could risk a certain amount of ‘mission drift’, increasing the chances that mindfulness does indeed become hijacked by capitalist and neo-liberal agendas in exactly the ways that many of the study participants seemed to wish to avoid. Here, the limitations of the present study become apparent, as gathering practitioner accounts of their motivations and aspirations is not quite the same as examining their conduct in situ, or the effects of their mindfulness teaching on client groups. More research is thus required in educational settings in the UK and other countries where mindfulness has a significant presence, to explore the socio-cultural outcomes of practitioner motivations and aspirations when translated into practice, including through the lens of student agency (e.g. see Hailwood, 2020).

## Notes

1. The use of Buddhist-inspired philosophies and practices to support wider social and political structures is not without historical precedent, such as during the reign of Ashoka (king of the Mauryan Empire from around 272–232 BC), whose promotion of non-violence and respect for authority as part of a broad and inclusive conception of *dharma*, was important for legitimising imperial governance and fostering social harmony and religious tolerance (see Laine, 2015).

2. The involvement of Zen Buddhism with Japanese militarism in the 1930s–40s, including the use of meditation for military training, led to several official apologies for what is now considered a contravention of Buddhist teachings on non-violence (see Jalon, 2003).

3.
https://mindfulnessinschools.org/

4.
https://www.youthmindfulness.org/

5.
https://www.thepresentcourses.org/

6.
https://myriadproject.org/

7.
https://www.mindfulschools.org/

8.
https://mindup.org/
